# Editorial: Endocrine and metabolic diseases – genetic impact and therapies

**DOI:** 10.3389/fendo.2022.1063167

**Published:** 2022-10-19

**Authors:** Isabelle Jéru, Bruno Fève, Ralf Jockers

**Affiliations:** ^1^ Sorbonne Université-Inserm Unité Mixte de Recherche en Santé (UMRS)_938, Centre de Recherche Saint-Antoine (CRSA), Paris, France; ^2^ Institute of Cardiometabolism and Nutrition (ICAN), Centre Hospitalier Universitaire (CHU) Pitié-Salpêtrière - Saint-Antoine, Assistance Publique-Hôpitaux de Paris (AP-HP), Paris, France; ^3^ Sorbonne Université, Assistance Publique-Hôpitaux de Paris, Hôpital Pitié-Salpêtrière, Département de Génétique Médicale, Département Médico-Universitaire (DMU) BioGeM, Paris, France; ^4^ Sorbonne Université, Assistance Publique-Hôpitaux de Paris (AP-HP), Hôpital Saint-Antoine, Service d’Endocrinologie, Diabétologie et Reproduction, Paris, France; ^5^ Université Paris Cité, Institut Cochin, Inserm, Centre National de la Recherche Scientifique (CNRS), Paris, France

**Keywords:** endocrinology, metabolic diseases, genetics, gene, therapy

Genetics plays an important role in endocrine and metabolic diseases. Despite many advances on genes implicated in these disorders and associated signaling pathways, there remain major challenges: an improved understanding of the heterogeneity of complex endocrine and metabolic disorders, especially obesity and diabetes, a better assessment of the risk associated with susceptibility genetic factors, and to improve personalized medicine. This Research Topic of *Frontiers in Endocrinology* explores through a number of complementary examples the latest work and emerging ideas related to the identification of genetic factors involved in endocrine and metabolic diseases, novel signaling pathways, translational research, and clinical applications.

The genetic contribution to diseases is usually evaluated by heritability, which is an estimate of how much of the disease susceptibility is attributable to genetic variations. The search for contributing genes in monogenic disorders started in the 1990’s, using large familial pedigrees and linkage analysis followed by candidate gene approaches. Many important discoveries have been made by applying this strategy in all fields of Mendelian genetics. Pathogenic variants, which segregate in families were found to be associated with highly penetrant disorders, thereby providing the first insights into the pathophysiology of the corresponding conditions. A second era in the identification of monogenic disorders occurred with the advent of exome sequencing, which revolutionized our understanding of rare diseases, uncovering causal rare variants for hundreds of these disorders. Besides disorders of Mendelian inheritance, the search for genetic variants that contribute to common multifactorial forms of endocrine and metabolic diseases started later, in the 2000’s, with the development of genome-wide association studies (GWAS). GWAS led to the identification of thousands of genetic loci associated with complex diseases. Nevertheless, identifying the causal gene(s) and/or variant(s) within each association locus remains an ongoing challenge. Increasing availability of high-throughput genome-scale technologies, advanced computational tools, comprehensive multi-omics databases should accelerate the translation of GWAS loci into meaningful biological markers. In certain conditions, such as diabetes or liver steatosis, polygenic scores have been proposed. A polygenic score represents an individual’s genetic susceptibility to develop a disease and is calculated by summing the number of susceptibility genetic variants, weighted by each variant’s effect size observed in a GWAS. Based on such genetic profiling, several direct-to-consumer genomic companies are already informing individuals about their risk and predisposition for a panel of common diseases and traits, including obesity and type 2 diabetes. Although these results have fueled expectations that genotype information could be used in clinical care for early diagnosis of high-risk individuals, the current quality of these predictions remains quite low (1). In between Mendelian genetics and GWAS, now appears the possibility to use data from UK Biobank, which contains detailed phenotypic data linked to medical records for approximately 500,000 participants, offering an unprecedented opportunity to evaluate the effect of rare variations on a broad collection of traits (2).

In addition to the genetic risk, the appearance and evolution of endocrine and metabolic disorders can be influenced by a number of environmental factors including socio-demographic, lifestyle, and clinical characteristics. Genes and environmental factors can also interact to modify our metabolism. Epigenetics, which corresponds to biochemical modifications influencing gene expression and activity, can regulate cellular processes and the whole-body physiology, as observed in type 2 diabetes (3). Another environmental factor that impacts the pathophysiology of endocrine disorders is the control of circadian rhythms (4). Endocrine organs release a variety of hormones in response to diurnal cycles of light/dark, fasting/feeding, and temperature changes (5). This is of clinical relevance since disruption of the circadian clock is linked to metabolic disease. The inflammatory state of a patient is also a determinant of the risk of type 2 diabetes and obesity. This is illustrated by the rapid development of a new field called ‘‘immunometabolism,’’ which studies the complex interactions between metabolic and inflammatory pathways in immune and metabolic tissues (5).

Personalized medicine is defined as the right treatment for the right person at the right time. To date, there are few examples of precision therapeutics. Patients with congenital leptin deficiency can benefit from leptin replacement to treat severe obesity (6). Patients with monogenic diabetes due to pathogenic variants in the genes encoding the KCNJ11 and ABCC8 potassium channel subunits are very responsive to sulfonylureas and do not need insulin therapy (7). Patients with monogenic diabetes due to *GCK* variants display a mild form of diabetes, stable over time, which does not require any treatment. The ambition to personalize all aspects of an individual’s management, including precision diagnosis, lifestyle and treatment, and prognosis has recently been underlined by international consortia in the case of diabetes (7). More comprehensive approaches comprising demographic, environmental, genetic, clinical, and biological markers will be needed to accurately predict who is at risk of a given condition. Classification in subtypes of heterogenous diseases, like obesity and type 2 diabetes, will also be a major challenge, and delineation of the diverse underlying biological mechanisms will be a pre-requisite to tailor prevention and treatment strategies.

## Author contributions

IJ, BF, RJ: wrote and validated the last version of the Editorial. All authors contributed to the article and approved the submitted version.

## Conflict of interest

The authors declare that the research was conducted in the absence of any commercial or financial relationships that could be construed as a potential conflict of interest.

## Publisher’s note

All claims expressed in this article are solely those of the authors and do not necessarily represent those of their affiliated organizations, or those of the publisher, the editors and the reviewers. Any product that may be evaluated in this article, or claim that may be made by its manufacturer, is not guaranteed or endorsed by the publisher.
